# Automated seaweed species classification using deep learning and large language models

**DOI:** 10.1038/s41598-026-63136-4

**Published:** 2026-07-23

**Authors:** Mahmoud Sami, Fayrouz Ahmed

**Affiliations:** Department of Marine Science, Faculty of Science, Suez Canal University, Ismailia, Egypt

**Keywords:** Seaweed classification, Deep learning, Convolutional neural networks, Claude AI, Large language models, Automated biodiversity monitoring, Data augmentation, Vision-language model, Human-in-the-loop, Marine technology, Computational biology and bioinformatics, Ecology, Ecology, Ocean sciences

## Abstract

Seaweeds are foundational components of marine ecosystems and hold significant value in global aquaculture. Accurate and efficient identification of seaweed species is critical for biodiversity monitoring, ecological research, and sustainable management. This study evaluates a dual-approach framework for automated seaweed classification. First, we implemented a standard convolutional neural network (CNN) based on the *EfficientNet-B0* architecture, utilizing transfer learning from ImageNet. The model was trained on an augmented dataset of 3,440 images derived from an original collection of 800 field images (stratified 80/10/10 split) covering 43 seaweed species. The CNN achieved a baseline classification accuracy of 89%. Second, we present a proof-of-concept study using a vision-language model (VLM), specifically *Claude 3.5 Sonnet*, to explore semantic reasoning in taxonomic classification. The VLM achieved a raw accuracy of 70%, which was further improved to an effective accuracy of 92% on a selected subset through a human-in-the-loop (HITL) validation system. While the CNN provides a robust and rapid classification tool, the VLM-HITL approach offers an interpretable semantic alternative for validating challenging specimens. This work contributes to the development of scalable, automated biodiversity monitoring tools for marine science.

## Introduction

Seaweeds, or marine macroalgae, are foundational to coastal marine ecosystems, contributing significantly to primary productivity and nutrient cycling^1,2^. The accurate identification of seaweed species is a cornerstone of marine biodiversity monitoring and conservation efforts^3^. Traditional species identification relies on morphological analysis by expert taxonomists—a process that is labor-intensive and limited by specialist availability^4^.

Recent advances in deep learning–based ecological monitoring have focused on improving detection performance under challenging environmental conditions through the integration of super-resolution techniques and enhanced YOLO-based frameworks. Super-resolution–assisted detection models have demonstrated notable improvements in UAV and underwater imagery by enhancing visual quality prior to object recognition^5^. In addition, more recent studies have combined semi-supervised learning with advanced YOLO architectures such as YOLOv10 and YOLOv13 to address the challenges of low-quality data and dynamic aquatic environments^26^ . Furthermore, the integration of amphibious unmanned aerial vehicle (UAV) systems with deep learning pipelines has enabled scalable and cost-effective ecological monitoring in shallow water ecosystems. Collectively, these studies highlight the importance of image enhancement and domain-specific adaptation in improving detection robustness in aquatic environments.

In parallel, recent developments in ecological image analysis have shifted toward more advanced representation learning paradigms beyond conventional convolutional neural networks, particularly Vision Transformers (ViTs) and Vision-Language Models (VLMs). These approaches are model global contextual relationships and incorporating semantic knowledge to enhance visual understanding^6–8^. Moreover, their zero-shot learning capability enables the recognition of unseen or rare species without task-specific retraining, offering significant advantages in biodiversity monitoring scenarios where data scarcity and taxonomic rarity are common challenges^8–10^. This study investigates a dual-approach framework for seaweed classification. We utilize a standard *EfficientNet-B0* CNN to establish a high-performance baseline and explore the potential of a VLM (*Claude 3.5 Sonnet*) as a proof-of-concept for interpretable, expert-assisted classification. The integration of a human-in-the-loop (HITL) system further bridges the gap between automated predictions and expert taxonomic validation.

## Materials and methods

### Dataset collection and preparation

An initial set of 800 original images was collected during field surveys conducted in the coastal waters of Hurghada, the Gulf of Suez, and Marsa Alam, Egypt (Red Sea coast). The dataset covers 43 species across three phyla: Chlorophyta, Phaeophyta, and Rhodophyta. The dataset exhibits an inherent class imbalance, with the number of images per species varying from approximately 14 to 21. This variation is attributable to ecological heterogeneity among habitats and species-specific abundance patterns (Table 1).

Taxonomic identification was performed through detailed morphological examination using standard taxonomic keys^11,^ ^2,12–15^;. Species identification followed established taxonomic criteria for marine macroalgae. The identifications were further validated by comparing the collected field images with reference specimens preserved in the Suez Canal University Herbarium.

Unlike web-scraped datasets, which may suffer from inconsistent labeling, variable image quality, and limited ecological context, the present dataset was constructed entirely from field-collected images under natural environmental conditions. This ensures higher ecological validity and a more realistic representation of species variability, making the dataset suitable for training and evaluating machine learning models for marine biodiversity applications. The combination of multi-site sampling, taxonomic validation, and natural habitat variability ensures that the dataset is representative for real-world ecological classification tasks.

#### Data augmentation and splitting

The original 800 images were expanded to 3,440 images using rotation (± 30°), flipping, brightness adjustments (0.8–1.2), and scaling (20% zoom). Augmentation parameters were applied adaptively, with more intensive transformations for minority classes, to mitigate class imbalance and ensure robust representation across all species. The dataset was split into 80% training, 10% validation, and 10% testing subsets using a stratified scheme, guaranteeing proportional representation of all 43 species in each subset.

**Table 1:** Taxonomic composition and dataset distribution of seaweed species.

No	Class	Species	No. of Images (Original)
1	Green algae (Chlorophyta)	*Caulerpa racemosa*	19
2		*Caulerpa serrulate*	18
3		*Chaetomorpha indica*	14
4		*Chaetomorpha linum*	19
5		*Cladophora prolifera*	18
6		*Cladophora rupestris*	21
7		*Bryopsis plumosa*	19
8		*Enteromorpha flexuosa*	20
9		*Enteromorpha intestinalis*	21
10		*Boergesenia forbesii*	18
11		*Codium decorticatum*	20
12		*Dictyosphaeria cavernosa*	20
13		*Valonia macrophysa*	21
14		*Valonia aegagropila*	18
15		*Halimeda tuna*	21
16		*Ulva lactuca*	20
17	Brown algae (Phaeophyceae)	*Feldmannia mitchellae*	19
18		*Cystoseira myrica*	17
19		*Hormophysa* sp	20
20		*Turbinaria ornata*	19
21		*Turbinaria turbinata*	18
22		*Sargassum* sp	19
23		*Padina boergesenii*	14
24		*Padina pavonica*	19
25		*Hormophysa cuneiformis*	20
26		*Hydroclathrus clathratus*	18
27		*Dictyota dichotoma*	17
28		*Colpomenia sinuosa*	19
29		*Lobophora variegata*	20
30	Red algae (Rhodophyta)	*Lithophyllum incrustans*	18
31		*Digenea simplex*	17
32		*Chondria dasyphylla*	19
33		*Jania rubens*	16
34		*Jania adhaerens*	19
35		*Laurencia obtuse*	17
36		*Laurencia papillosa*	20
37		*Hypnea cornuta*	19
38		*Actinotrichia fragilis*	18
39		*Galaxura rugosa*	16
40		*Palisada perforate*	19
41		*Ganonema farinosum*	18
42		*Peyssonnelia rubra*	20
43		*Gracilaria gracilis*	18

### CNN model development

The CNN utilized the EfficientNet-B0 architecture with transfer learning (ImageNet pretrained weights). This architecture was selected for its efficiency and state-of-the-art performance in biological classification ^,16^. The model was trained to recognize key morphological features, such as branching patterns (e.g., dichotomous vs. pinnate), thallus texture (e.g., smooth, rugose), the presence of air bladders (pneumatocysts), and overall structural complexity^17,18^.

#### Architecture and implementation

The model was implemented in TensorFlow/Keras. The top layer of the EfficientNet-B0 base was replaced with a Global Average Pooling layer followed by a fully connected Dense layer with Softmax activation for 43-class classification. This design enabled the model to leverage high-level feature representations learned from ImageNet while adapting to the specific characteristics of the seaweed dataset.

#### Training protocol

The CNN model was trained for a total of 35 epochs, with early stopping applied based on validation loss (patience = 5) to prevent overfitting and ensure optimal generalization. Transfer learning was implemented using a two-stage strategy: initially, the base layers of EfficientNet-B0 were frozen to preserve general feature representations learned from large-scale ImageNet data, while only the classification head was trained. In the second stage, selected upper convolutional layers were gradually unfrozen to enable fine-tuning and adaptation to domain-specific features of seaweed morphology. This approach improved the model’s ability to capture fine-grained differences among visually similar species while maintaining stability in training. The complete training configuration, including optimizer settings, learning rate, loss function, batch size, and other hyperparameters, is summarized in Table 2.

**Table 2:** CNN training protocol parameters.

Parameter	Value
Architecture	EfficientNet-B0
Pretrained Weights	ImageNet
Optimizer	Adam (1 × 10⁻³)
Batch Size	32
Loss Function	Categorical Cross-Entropy
Early Stopping	Patience = 5 (based on validation loss)

### Claude AI vision-language model (VLM) development

To explore an alternative classification paradigm beyond conventional convolutional neural networks, we implemented a vision–language model (VLM) based on the multimodal capabilities of Claude 3.5 Sonnet accessed via the Anthropic API. Unlike CNN-based models that rely primarily on pixel-level pattern recognition, VLMs integrate visual understanding with semantic reasoning, enabling the model to interpret visual features in conjunction with contextual knowledge. This paradigm aligns with recent developments in multimodal AI systems capable of performing structured reasoning over visual inputs and generating interpretable outputs^8,19^.

In this framework, the model receives an input image together with a constrained classification prompt that limits the possible outputs to a predefined list of seaweed taxa. The objective of this approach was not only to assess classification performance but also to evaluate the interpretability and reasoning capabilities of a large vision–language model in a marine biodiversity monitoring context.

To ensure a fair and reproducible evaluation, a total of 100 test images were randomly selected from the held-out test set, ensuring representative coverage across the 43 seaweed species. The reported VLM performance corresponds to raw model predictions without any human intervention, reflecting the standalone capability of the model.

#### Technical configuration and prompt engineering

To ensure reproducibility and controlled inference behavior, the Claude VLM was queried using a structured prompt-engineering strategy. Each classification request included a system prompt designed to emulate expert taxonomic reasoning while constraining the output to the predefined species list.

The system prompt used for each query was structured as follows:

“You are an expert marine taxonomist. Analyze the following image and identify the seaweed species from this list: [List of 43 species]. Provide your answer in JSON format with the fields ‘species_name’, ‘confidence’, and ‘reasoning’.”

This structured output format ensured that the model returned standardized responses containing the predicted species name, an associated confidence score, and an explanatory reasoning component describing the morphological features influencing the decision.

The Claude API was configured with controlled inference parameters to reduce stochastic variation and improve reproducibility. Specifically, the temperature parameter was set to 0 to enforce deterministic outputs, while the top-p parameter was set to 0.9 to maintain limited probabilistic diversity within the sampling distribution. The maximum token length was limited to 512 tokens to allow sufficient space for structured reasoning without excessive response length.

Model responses were automatically parsed using a Python-based regular expression script, which extracted the predicted species label, confidence score, and explanatory reasoning from the returned JSON structure. These parsed outputs were subsequently used for evaluation and integration into the validation workflow.

#### Human-in-the-loop (HITL) validation system

To improve reliability and interpretability, a Human-in-the-Loop (HITL) validation framework was integrated into the Claude AI classification pipeline. Human-guided validation mechanisms are increasingly recognized as essential components of Explainable Artificial Intelligence (XAI), particularly in ecological applications where classification errors may have significant implications for biodiversity monitoring and conservation^20–23^.

Within this workflow, the Claude model generates a predicted species label, confidence score, and explanatory rationale for each input image. Predictions with confidence scores below a predefined threshold (confidence < 0.7) are automatically flagged for expert review. These cases typically correspond to visually ambiguous taxa or morphologically similar or underrepresented species.

During the validation stage, a marine biologist with taxonomic expertise reviews the flagged predictions, verifies the classification, and corrects labels when necessary. The validated outputs are stored in a structured SQL database, enabling systematic recording of expert corrections.

This process establishes a continuous learning loop, where expert feedback can be used to guide future prompt refinement and potential model optimization. Such iterative human–AI collaboration is particularly valuable for addressing the long-tail distribution of rare marine species and improving robustness in fine-grained ecological classification tasks^23^.

### Model deployment and accessibility

To enhance the practical utility and accessibility of the proposed framework, the trained convolutional neural network (CNN) model was deployed as an interactive web application using Hugging Face Spaces. This platform was selected due to its ease of access and capability to host machine learning models without requiring local installation or specialized computational resources^20^. The application was implemented using the Gradio library, providing an intuitive interface that enables users to upload seaweed images and obtain real-time classification results.

The system returns the top-5 predicted species along with their corresponding confidence scores, offering interpretable outputs that can facilitate marine biodiversity monitoring and environmental education applications.

For reproducibility, the deployment includes the trained Keras model file (best_seaweed_aug_model.keras), class label mappings (class_names.json), and all required dependencies.

The application is publicly accessible at: https://huggingface.co/spaces/Mahmoudsami/Seaweed_Identifier.

The Claude AI model is maintained as a “Claude AI Artifact,” representing a combination of the specific model version, optimized prompts, and the HITL protocol. It is accessible via API for integrated applications and is available at: Claude AI Artifact (Seaweed Identifier: AI-Powered Marine Algae Recognition Tool | Claude).

### Evaluation metrics

The performance of both models was quantitatively assessed using standard classification metrics:

- Accuracy: The proportion of correctly classified images. It is calculated as (True Positives + True Negatives)/(Total Samples).
- Top-5 Accuracy: The proportion of images where the correct class is among the top five predictions. This metric is particularly relevant for practical applications where the model serves as a decision-support tool for experts.
- F1-Score: The harmonic mean of precision and recall. It is a robust metric for evaluating performance on imbalanced datasets. We report the macro-averaged F1-score, which calculates the metric independently for each class and then averages them.
- Confusion Matrix: A matrix used to visualize the performance of the classification model. Each row represents the instances in an actual class, while each column represents the instances in a predicted class. This is crucial for identifying specific inter-species misclassifications.

## Results

### CNN model performance

The CNN model demonstrated strong performance on the independent stratified test set, indicating effective generalization to unseen seaweed images. This performance is attributed to the combined effect of transfer learning and an extensive data augmentation strategy applied during training. The overall evaluation metrics are summarized in Table 3.

**Table 3:** Performance Metrics of the CNN model on the test set.

Metric	Value (%)
Accuracy	89%
Top-5 Accuracy	95%
Macro F1-Score	0.88

**Fig. 1:**
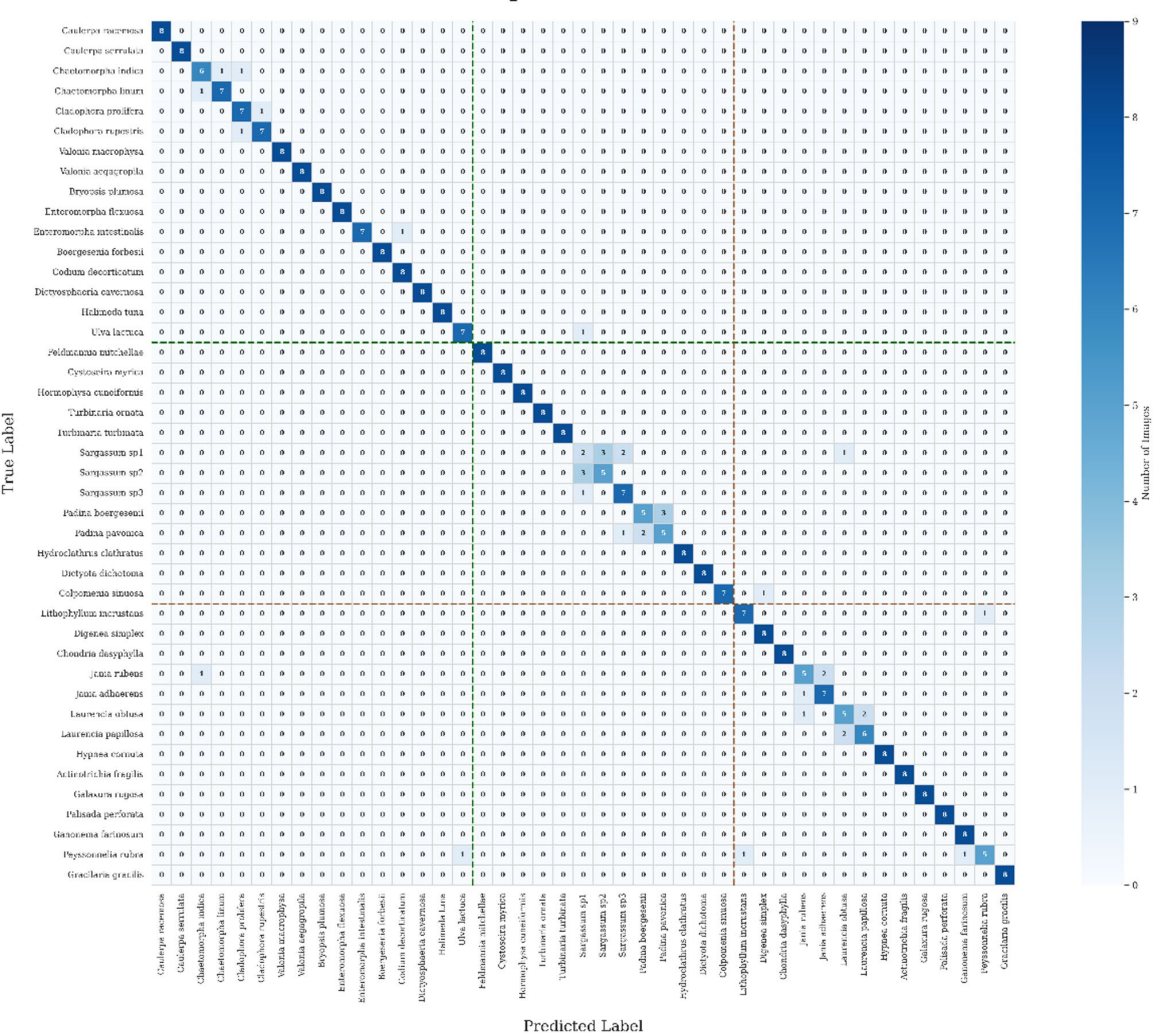
Confusion matrix illustrates the classification performance of the proposed CNN model across 43 seaweed species in the test dataset.

To further interpret model behavior, a detailed analysis of the confusion matrix (Fig. 1) was conducted. The results indicate that most misclassifications occur between morphologically similar species within the same genus, particularly among *Sargassum* species and between *Padina pavonica* and *Padina boergesenii*. These errors are primarily attributed to high intra-genus morphological similarity in thallus structure and texture. In contrast, misclassifications across major algal divisions (Chlorophyta, Phaeophyta, and Rhodophyta) were rare, demonstrating that the model successfully learned robust high-level taxonomic features. Overall, these findings suggest that while the model performs reliably at the phylum and genus level, fine-grained species-level discrimination remains the primary challenge in visually similar taxa.

### Claude AI VLM performance and HITL enhancement

The Claude AI vision–language model (VLM) achieved a baseline classification accuracy of 70% when evaluated on the held-out seaweed image subset using the constrained species list described in the Methods section. Considering that the classification task involved 43 candidate species, this performance represents a substantial improvement over the theoretical random baseline accuracy of approximately 2.3%, corresponding to nearly a 30-fold increase in classification performance.

Consistent with the Human-in-the-Loop (HITL) validation framework, predictions associated with low confidence scores (confidence < 0.7) were automatically flagged for expert review. In a validation trial involving 100 flagged images, the marine taxonomist examined the model predictions and corrected the species labels where necessary. Within this reviewed subset, 92% of the initial model errors were successfully corrected through expert intervention.

Following the integration of these expert corrections, the combined VLM + HITL workflow achieved a final effective accuracy of approximately 96% on the reviewed subset, demonstrating the substantial benefit of incorporating domain expertise into AI-assisted ecological classification pipelines.

These findings highlight the complementary strengths of multimodal AI models and expert validation, particularly for datasets containing morphologically similar taxa or species with high intra-specific variability^30^.

## Discussion

This study demonstrates the effectiveness of two complementary artificial intelligence approaches for automated seaweed species classification. The high accuracy (89%) achieved by the CNN model highlights the capability of deep learning when combined with a carefully curated and augmented dataset. Expanding the original 800 field images to more than 3,000 training samples enabled the model to learn robust and generalizable visual features, consistent with best practices in computer vision–based ecological classification^31^. The performance of our EfficientNet-based model is comparable with recent studies in related ecological domains. For example, seaweed classification using EfficientNet has reported accuracies around 89.55% (Çiçek et al., 2025), while fish species classification using Swin Transformer architectures has achieved accuracies exceeding 98% under controlled datasets^6^. Although the accuracy obtained in the present study is competitive, these comparisons highlight opportunities for future improvements through the adoption of more advanced architectures such as Vision Transformers (ViTs)^32^.

A notable aspect of this study is the integration of two distinct AI paradigms within a single analytical framework. Specifically, the approach combines a specialized convolutional neural network (EfficientNet-B0) with a general-purpose vision–language model (Claude 3.5), linked through a Human-in-the-Loop (HITL) validation process. This dual strategy allows the CNN to perform high-throughput visual classification, while the VLM contributes semantic interpretation and explanatory reasoning that can support expert validation. Compared with purely automated systems, incorporating expert feedback provides a practical mechanism for addressing challenges associated with rare or visually ambiguous taxa, a phenomenon often described as the *long-tail problem* in ecological datasets^20^.

The Claude AI–based vision–language model represents a different classification paradigm based on semantic interpretation rather than solely on learned pixel patterns. A key trade-off therefore emerges between the CNN’s highly specialized feature extraction and the VLM’s broader contextual reasoning capabilities. While the CNN achieved higher raw classification accuracy, the VLM demonstrated the ability to provide descriptive reasoning associated with its predictions, referencing morphological features that support species identification. This characteristic aligns with the objectives of Explainable Artificial Intelligence (XAI), which aims to improve transparency and interpretability in AI-assisted scientific workflows^19,22^.

In contrast to recent detection-focused approaches such as SFD-YOLO (2025), which prioritize real-time object detection, the proposed framework emphasizes fine-grained taxonomic classification and interpretability. This makes it particularly suitable for biodiversity monitoring applications where species-level accuracy and expert validation are essential.

The integration of the HITL framework further strengthens the analytical pipeline by enabling expert verification of uncertain predictions. This collaborative interaction between AI models and domain specialists is especially valuable in ecological studies where dataset imbalance and morphological variability can challenge purely automated systems^1,21^.

The deployment of the CNN model through Hugging Face Spaces enhances accessibility by providing a user-friendly interface for researchers, educators, and citizen scientists. Open-access AI tools can support large-scale ecological data collection and contribute to improved biodiversity monitoring efforts across different regions^33^. This participatory approach may also facilitate a better understanding of seaweed distribution dynamics and their responses to environmental changes^34^.Overall, the comparative analysis of the two models highlights a clear trade-off between speed and raw accuracy on one hand, and interpretability and expert-assisted validation on the other. Rather than representing competing approaches, the CNN and VLM–HITL components can be viewed as complementary tools within an integrated framework for marine biodiversity monitoring^35^.

Despite the promising results obtained in this study, several limitations should be acknowledged. First, the dataset was derived from approximately 800 original field images collected from the western coast of the northern Red Sea, which may limit the generalizability of the models to other geographic regions or environmental conditions. Although data augmentation expanded the training dataset to over 3,000 images, the representation of some rare species remained limited, which may affect classification performance for underrepresented taxa. In addition, the evaluation of the vision–language model (VLM) component should be considered preliminary, as its performance was assessed on a relatively small subset and within a constrained prompting framework. Further validation using larger, independently collected datasets and standardized benchmarking protocols will be necessary to rigorously assess the robustness and scalability of the VLM-based approach. Addressing these limitations in future work will help improve the reliability and broader applicability of AI-assisted seaweed classification systems for marine biodiversity monitoring.

## Conclusion

This study presents a dual-approach framework for seaweed classification across 43 species, achieving 89% accuracy using an EfficientNet-B0-based CNN trained on a curated and augmented dataset. The Claude 3.5 Sonnet vision–language model showed promising preliminary capability for semantic validation with 70% baseline performance, while the Human-in-the-Loop (HITL) framework improved prediction reliability for challenging cases. Overall, the results demonstrate the complementary role of high-accuracy deep learning and interpretable multimodal AI in marine biodiversity assessment. Future work should expand the dataset to broader geographic regions and develop domain-specific vision–language models to enhance scalability and reduce reliance on manual taxonomic validation.

## Data Availability

The datasets used and/or analyzed during the current study available from the corresponding author on reasonable request.

## Code availability

The training, evaluation, and inference code is publicly available at:

https://huggingface.co/spaces/Mahmoudsami/Seaweed_Identifier.

The repository includes the full training pipeline (train_model.py), deployed application (app.py), trained EfficientNet-B0 model, class labels, and all required dependencies necessary to reproduce the prediction workflow and evaluate the proposed framework.
